# The Human Virome Protein Cluster Database (HVPC): A Human Viral Metagenomic Database for Diversity and Function Annotation

**DOI:** 10.3389/fmicb.2018.01110

**Published:** 2018-05-29

**Authors:** Ali H. A. Elbehery, Judith Feichtmayer, Dave Singh, Christian Griebler, Li Deng

**Affiliations:** ^1^Institute of Virology, Helmholtz Zentrum München – Deutsches Forschungszentrum für Gesundheit und Umwelt, Oberschleißheim, Germany; ^2^Institute of Groundwater Ecology, Helmholtz Zentrum München – Deutsches Forschungszentrum für Gesundheit und Umwelt, Oberschleißheim, Germany; ^3^EvA Consortium, Manchester, United Kingdom; ^4^Medicines Evaluation Unit, University Hospital of South Manchester Foundation Trust, University of Manchester, Manchester, United Kingdom

**Keywords:** viral metagenomics, human virome, lung virome, protein cluster, bronchoalveolar lavage, chronic obstructive pulmonary disease

## Abstract

Human virome, including those of bacteria (bacteriophages) have received an increasing attention recently, owing to the rapid developments in human microbiome research and the awareness of the far-reaching influence of microbiomes on health and disease. Nevertheless, human viromes are still underrepresented in literature making viruses a virtually untapped resource of diversity, functional and physiological information. Here we present the human virome protein cluster database as an effort to improve functional annotation and characterization of human viromes. The database was built out of hundreds of virome datasets from six different body sites. We also show the utility of this database through its use for the characterization of three bronchoalveolar lavage (BAL) viromes from one healthy control in addition to one moderate and one severe chronic obstructive pulmonary disease (COPD) patients. The use of the database allowed for a better functional annotation, which were otherwise poorly characterized when limited to annotation using sequences from full-length viral genomes. In addition, our BAL samples gave a first insight into viral communities of COPD patients and confirm a state of dysbiosis for viruses that increases with disease progression. Moreover, they shed light on the potential role of phages in the horizontal gene transfer of bacterial virulence factors, a phenomenon that highlights a possible contribution of phages to etiopathology.

## Introduction

The human virome is comprised of all viruses found on or in humans, including eukaryotic and prokaryotic viruses (Wylie et al., 2012). The term virome is also used to refer to viral metagenomes, which are the overall collection of genetic material isolated from viral like particles (VLPs) in a given environment (Haynes and Rohwer, 2011). Recently, the human microbiome field has witnessed a big revolution following the unprecedented advances in sequencing technologies (Rescigno, 2017). Yet, the human virome research is still lacking behind when compared to studies addressing the prokaryotic component of the human microbiome (Zou et al., 2016). It is not surprising to know that out of 920 million dollar fund for microbiome research in the United States between 2012 and 2014, only 3% was used for studying viral communities (Stulberg et al., 2016). In light of these limited virome studies, it is generally believed that bacteriophages constitute the major fraction of the human virome (Haynes and Rohwer, 2011). In addition to their role in controlling the population dynamics of their hosts, bacteriophages can support their hosts with new phenotypes through horizontal gene transfer. These roles can potentially influence human health through modifying the structure of the bacterial community or imparting novel pathogenicity attributes to their hosts (Haynes and Rohwer, 2011; Wylie et al., 2012). Therefore, the abundance and diversity of detected bacteriophages differ considerably between health and disease (Willner et al., 2009). Indeed, many studies indicated that changes in the abundance of bacterial species or imbalance of their dynamic equilibrium is linked to several human disorders (Ley et al., 2006; Hilty et al., 2010; Huffnagle, 2010). Contribution of phages to this shift may be due to their potential involvement in influencing the microbial composition by altering the ratio of symbionts to pathobionts (Mills et al., 2013). Bacteriophages may affect human health as they influence bacterial population structures or virulence (Waldor and Mekalanos, 1996). Thus phages may be important effectors and indicators of human health and disease (Wylie et al., 2012). Similarly, eukaryotic viruses are detected in both healthy and diseased subjects, despite being thought of as always pathogenic. The presence of these eukaryotic viruses, especially in healthy or asymptomatic subjects is suggested to be (i) transient due to environmental influence, (ii) a low grade infection controlled by the immune system, or (iii) some sort of commensalism (Okamoto, 2009; Willner et al., 2009; Haynes and Rohwer, 2011).

Estimates of the total number of viruses in and on the human body depend on the method of calculation. Based on the normal virus to host ratio of 10:1 usually detected in the environment, the overall number of viruses would be estimated to be 10^15^ viruses. In contrast, based on the experimentally detected viral counts in different body tissues, the number of viruses in the human body is likely to be around 3 × 10^12^ (Haynes and Rohwer, 2011). However, this number is probably an underestimation because direct counts were mostly determined using epifluorescence microscopy, which typically only considers double-stranded DNA viruses (Hennes and Suttle, 1995). Concerning diversity, it is projected that viral diversity in the human body is way lower than that in the environment. Haynes and Rohwer (2011) estimated that the number of viral genotypes in a healthy human should be a round 1,500, while in one kilogram of marine sediment, they suggest that viral genotypes should be not less than 10,000 and probably up to one million.

Despite growing interest in recent years, studying viromes remains a challenging endeavor due to several reasons, including the scarcity of viral genomic material (compared to microbial and human nucleic acid fraction) due to the small genome sizes of viruses and their low abundance in some cases (Wylie et al., 2012). In a microbial community only 2–5% of the total DNA is generally of viral origin (Reyes et al., 2010; Minot et al., 2011). Additionally, no conserved gene regions applicable for all viral types have been identified so far. Moreover, many viruses have not been characterized yet and are not included in viral databases (Woolhouse et al., 2008). These facts impede straightforward contig assemblages as well as functional annotation of viral genomes and metagenomes.

In this study, we present the human virome protein cluster (HVPC) database as an effort to improve functional annotation and characterization of human viromes. The database was built out of hundreds of virome datasets from six different body sites. We also show the utility of this database through its use for the characterization of three bronchoalveolar lavage (BAL) viromes from one healthy control in addition to one moderate and one severe chronic obstructive pulmonary disease (COPD) patients. In fact, lung viromes are generally poorly studied with only few reports from cystic fibrosis (CF) patients (Willner et al., 2009, 2012) or lung transplant recipients (Young et al., 2015; Abbas et al., 2017). To our knowledge, there are no previous studies on COPD viromes. Indeed, the use of the database not only did it allow for a better functional annotation, but also improved diversity analysis of the BAL viromes.

## Materials and Methods

### Dadabase Construction

#### Publicly Available Metagenomic Sequences

We downloaded a total of 245 virome sequences (**Supplementary Table S1**): 181 from The National Center for Biotechnology Information (NCBI) sequence read archive (SRA^1^) and 64 from the Metagenomic Analysis Server (MG-RAST^2^) (Meyer et al., 2008). Viromes were from gut (fecal), lung, mouth, oropharynx, skin and urine. Selected viromes were mostly from healthy individuals except in the case of lung viromes, which were from transplanted lungs or HIV-positive patients (Young et al., 2015).

#### Sequence Quality Control

TagCleaner v0.12 (Schmieder et al., 2010) was used to predict and trim tag and/or adapter sequences. In the case of paired-end Illumina sequences, AdapterRemoval v2.1.7 (Lindgreen, 2012) was used, instead. PRINSEQ-lite v0.20.4 (Schmieder and Edwards, 2011b) was used for quality filtering with following parameters: “-trim_qual_left 15 -trim_qual_right 15 -trim_qual_type mean -trim_qual_window 2 -lc_threshold 50 -lc_method entropy -derep 12345 -noniupac -ns_max_n 2 -min_qual_mean 20 -min_len 50 -max_len 800.” Reads with an average Phred score of less than 20 were filtered out. Viromes were decontaminated from human-related sequences using DeconSeq v0.4.3 (Schmieder and Edwards, 2011a) and human reference genome GRCh38 with default parameters. Deconseq was also used to remove reported spiked viral sequences e.g., PhiX174.

#### Sequence Assembly, ORF Calling and Clustering

Sequences were assembled using Newbler v2.9 (Roche) with default parameters. For Illumina sequences, we used MEGAHIT v1.0.3 (Li et al., 2015) with meta-sensitive preset mode and minimum contig length of 180. Open reading frames (ORFs) were predicted from both contigs and reads using Prodigal v2.6.3 (Hyatt et al., 2010) in its metagenomics mode. Predicted proteins were then filtered to keep only non-redundant sequences of at least 60 amino acids length. ORFs from publicly available sequences were then clustered using CD-HIT v4.6 (Li et al., 2001) with 60% identity, 80% coverage and the following parameters: “-g 1 -n 4 -d 0” to give rise to the HVPC database.

#### HVPC Database Functional Annotation

Representative ORFs from each cluster were annotated using four different methods: (i) hmmsearch against hmm models from the Prokaryotic Virus Orthologous Groups (pVOG, downloaded on March 28, 2017) (Grazziotin et al., 2017) with a threshold of 1 × 10^-3^ *e*-value and a bit-score of 50, (ii) hmmsearch versus Pfam30.0 release (Finn et al., 2014) (downloaded on December 28, 2016) with an *e*-value of 1 × 10^-3^ and a bit-score of 50; gene ontology was inferred based on the best hit Pfam accession through mapping to pfam2go (http://geneontology.org/external2go/pfam2go, version date: November 26, 2016, downloaded on: January 9, 2017) (Mitchell et al., 2015), (iii) comparison against FIGfams protein families (Meyer et al., 2009) through the FIGfams Server using svr_assign_using_figfams command line service from the SEED Servers (Aziz et al., 2012) with default parameters; SEED subsystems and categories were determined using svr_roles_to_subsys command line service from the SEED severs (Aziz et al., 2012) and (iv) BLASTp (Altschul et al., 1990) versus the non-redundant protein database from NCBI (nr, ftp://ftp.ncbi.nlm.nih.gov/blast/db/, downloaded on April 18, 2017) with an *e*-value of no more than 1 × 10^-3^ and a bit-score of at least 50.

### Experimental Methods

#### Bronchoalveolar Lavage (BAL) Samples Ethics Statement and Approval of the Institutional Review Board

The emphysema versus airway disease (EvA) study is an EU-funded project (# 200506) under the Seventh Framework Program (FP7). During visit one, candidates willing to participate have the opportunity to discuss all aspects and informed consent is obtained in line with the Declaration of Helsinki and based on approval by the local ethics committees and those samples analyzed by this manuscript were covered by ethics approval 08/H0402/19 (the NHS National Research Ethics Service, Nottingham, United Kingdom).

#### Sampling

The recruitment of patients and non-COPD controls was undertaken over a 3-year period (February 2009 to March 2012) for clinical examinations, computed tomography and lung function analysis. For assessing the viral composition in the lungs of patients and a non-COPD control, only the samples resulting from a BAL were used. The lavage was performed in the upper left lobe with a total volume of 150 ml sterile, pyrogen-free 37°C saline (Ziegler-Heitbrock et al., 2012).

#### DNA Extraction for Viral Metagenomics Sequencing

Four ml of each BAL sample was filtered [0.22 μm pore size (Millex-GP, Merck-Millipore, Billerica, MA, United States)] and DNase I (Roche, Switzerland)-treated in order to exclude contamination with prokaryotic or eukaryotic cell mass and DNA. DNA extraction was conducted afterward as described by Henn et al. (2010). A 16S rRNA PCR was done to check on the purity of viral samples and confirm freedom from bacterial contamination (universal primers Ba27 forward (5′-AGA GTT TGA TCM TGG CTC AG-3′) and Ba907 reverse (5′-CCG TCA ATT CMT TTR AGT TT-3′) (Lane, 1991). The following cycling conditions were used: 94°C for 5 min, (30 s at 94°C, 30 s at 52°C, 1 min at 70°C) × 29 cycles and 5 min at 70°C.

#### Amplification Step

As 454 pyrosequencing requires 3–5 μg of DNA for library preparation, but the average yield per sample was around 20 ng, an amplification step was required. We carried out the linker amplification method as described by Duhaime et al. (2012). In short, the extracted DNA was sheared into 500 bp fragments (Covaris E220, Woburn, MA, United States), end-repaired (End-It DNA End-Repair Kit, Epicentre Madison, WI, United States) and oligonucleotide linkers were ligated (Fast-Link DNA Ligations Kit, Epicentre). After a size selection (520–650 bp) using Pippin Prep (Sage Science, Beverly, MA, United States), a small-scale Polymerase chain reaction (PCR) titration with barcoded phos-A-PCR primer (5′-p-CCACACAGATCACGAAGCATAC) was performed to determine the optimal cycle number resulting in a high molecular weight DNA product accompanied by a low heteroduplex formation. 25 μl PfuUltra II Hotstart PCR Master Mix (Agilent, Santa Clara, CA, United States), 2 μl 10 μM barcoded primer, 20 μl nuclease-free water and 3 μl DNA template. A PCR cycler (Eppendorf) was used to run the following program: 2 min at 95°C (0.5 min at 95°C, 1 min at 60°C, 1.5 min at 72°C) × 17, 20, 23, 26, 28 cycles, 10 min at 72°C. After a large scale PCR, the quantity of the amplified DNA was determined using Quant-iT PicoGreen dsDNA Assay Kit (Invitrogen, Carlsbad, CA, United States). For a quality assessment 1–2 μl were run in a 1.2% agarose gel in 1x TAE as well as on a DNA 7500 Bioanalyzer Chip (Agilent). In total 23 lavage fluid samples from COPD patients and controls were provided from the EvA consortium, three field good enough DNA for viral metagenomic sequencing and were sequenced in the follow step.

#### Sequencing Technique 454

The preparation of the samples for 454 pyrosequencing with the GS FLX+ Instrument (Roche, Basel, Switzerland) followed the provided manuals. Only one of four lanes was used for the BAL samples with all samples barcoded and pooled equally.

### Bioinformatic Analyses of the BAL Samples

#### Sequence Quality Filtering and Assembly

Both quality filtering and assembly of sequences were done the same way they were done for the publicly available metagenomic sequences (Please refer to the section Database Construction).

#### Functional Annotation and Cross-Comparison of BAL Samples

A BLAST database was created from the representative ORFs of all clusters of the HVPC database. BAL ORFs were functionally annotated through alignment to the HVPC blast database using BLASTp (Altschul et al., 1990) with a threshold *e*-value of 1 × 10^-3^ and a bit-score of at least 50. For comparison, BAL ORFs were also aligned to protein coding sequences from all RefSeq and non-RefSeq complete viral genomes downloaded from NCBI (https://www.ncbi.nlm.nih.gov/ on May 10, 2017) as described by Grazziotin et al. (2017). The alignment was done using BLASTp (Altschul et al., 1990) with an *e*-value of not more than 1 × 10^-3^ and a bit-score of at least 50. For functional annotation, if the best hit is a hypothetical protein or of unknown function, the next hit was used as long as it fulfills the threshold criteria.

The three BAL viromes were also compared in a reference-independent manner using crAss (Dutilh et al., 2012). All high quality reads from the three samples were cross assembled using Newbler v2.9 (Roche) and used as an input for crAss.

#### Taxonomic Assignment of BAL Samples

A protein blast database was created from protein coding sequences of all RefSeq and non-RefSeq complete viral genomes downloaded from NCBI (https://www.ncbi.nlm.nih.gov/ on May 10, 2017) as described by Grazziotin et al. (2017). Only DNA viruses were selected by asserting that the “molecule type” feature in the genbank record of each genome is equal to DNA. Protein coding sequences from BAL samples were aligned to the created blast database using a threshold *e*-value of not more than 1 × 10^-3^ and a bit-score of at least 50. Taxonomy ids of best hits were mapped to NCBI taxonomy (downloaded from ftp://ftp.ncbi.nih.gov/pub/taxonomy on June 11, 2017) to obtain the full lineage.

#### Functional and Taxonomic Diversity Analysis of BAL Samples

Irrespective of annotation, alignment of BAL samples to the HVPC blast database was used as a measure of functional diversity in a sample. HVPC hit counts were prepared, then diversity indices and rarefaction plots were generated in R v3.4.0 (R Core Team, 2017) using vegan package v.2.4-4 (Oksanen et al., 2017). Taxonomy rarefaction analysis was similarly performed.

#### Virome-Borne Virulence Factors

A protein BLAST database was created from sequences downloaded from the core Virulence Factor Database (VFDB^3^) created by the MOH Key Laboratory of Systems Biology of Pathogens, Institute of Pathogen Biology, the Chinese Academy of Medical Sciences & Peking Union Medical College. This database includes sequences from experimentally verified virulence factors from 74 different genera of bacterial pathogens. All high quality reads from BAL samples were compared to this database using BLASTx (*e*-value ≤ 0.001, % identity ≥ 75%, alignment length ≥ 25 amino acids) to look for potential virulence factors harbored by each BAL virome. Random subsampling (sample size: 2000 reads) was performed 10,000 times; each time looking for and counting VFDB hits for each virome. One-way analysis of variance (ANOVA) was performed followed by Dunnett’s *post hoc* test to assess statistical significant difference between the means of virulence factor abundance among all BAL sample pairs.

## Results

### The HVPC Database

#### Description

**FIGURE 1:**
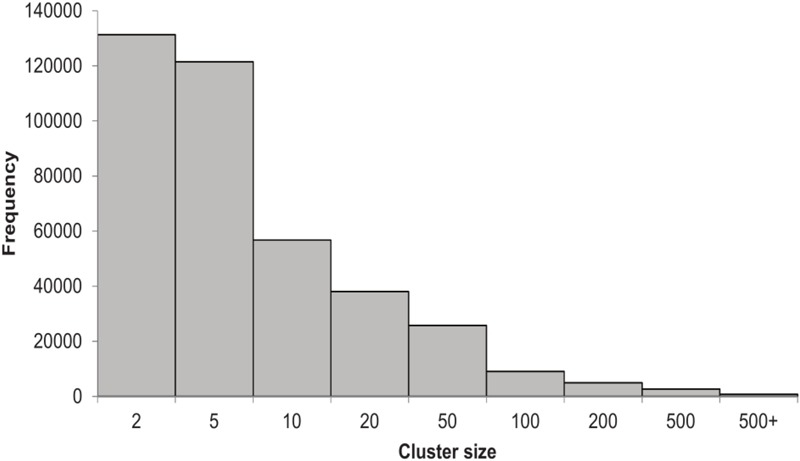
Histogram showing the number of clusters of the HVPC per cluster size.

We built the HVPC from 244 human virome datasets from 13 previously published studies in addition to one unpublished lung virome from NCBI’s SRA (**Supplementary Table S1**). In total, we used more than 86 million high quality sequence reads, constituting more than 12 terabases (**Supplementary Table S2**). Sequence reads were assembled separately and ORFs predicted from both contigs and reads. A total of 6,134,902 non-redundant ORFs (>60 amino acids in length) were called. These ORFs were clustered giving rise to 390,917 clusters of two or more members, in addition to 535,902 singletons (8.7% of ORFs). As for cluster size, which refers to the number of ORFs per cluster, most of the HVPC clusters (∼65%) are of two to five members (**Figure 1**). Clusters with members of 6–10, 11–20 and 21–50 represent 15, 10, and 7% of clusters, respectively. Only 4% of clusters have more than 50 members. The largest HVPC cluster has 72,340 ORFs.

**FIGURE 2:**
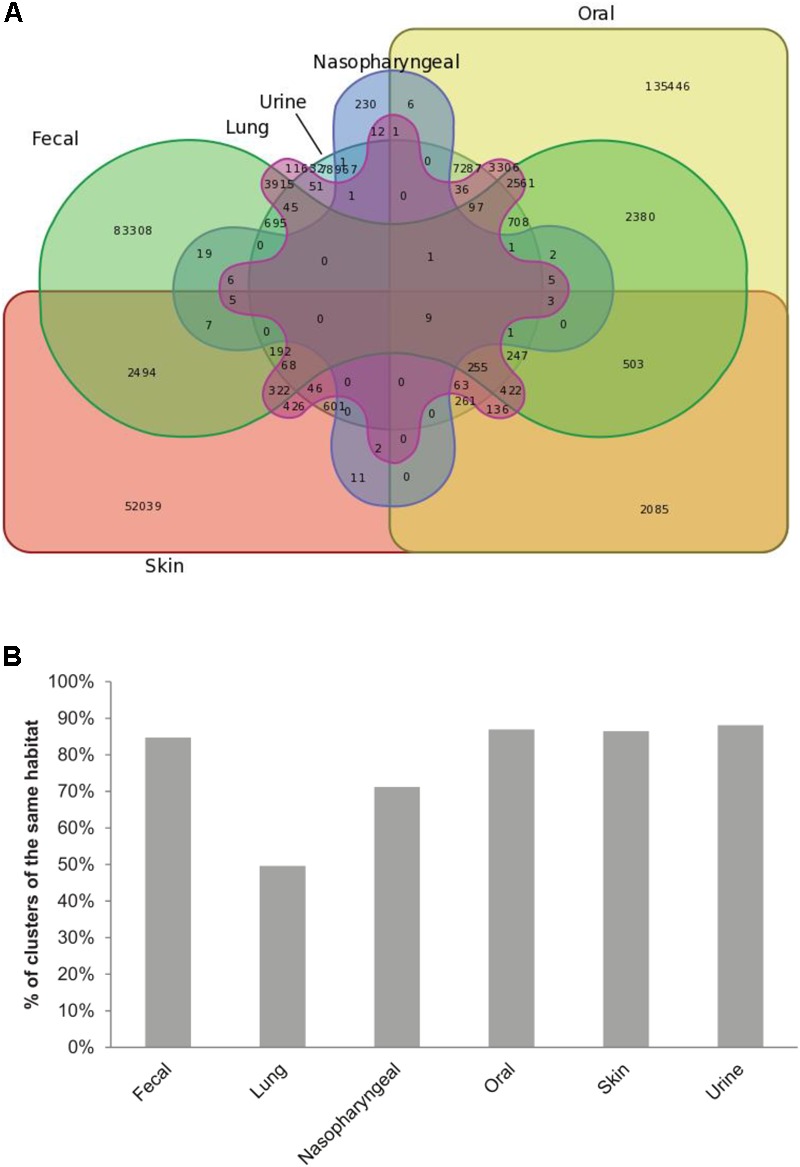
ORFs from the same body site cluster together. **(A)** Venn diagram showing the distribution of HVPC clusters between body sites. **(B)** Bar chart showing, for each body site, the percent of clusters made out of ORFs from this site alone compared to the total number of clusters containing at least one ORF from the same site.

Analysis of the origin of ORFs included in each cluster showed that ORFs from the same body site tend to cluster together (**Figure 2**). Clusters made solely out of ORFs from the same body site represent 93% of clusters (361,622 clusters). For most body sites, the number of clusters made exclusively from ORFs of a given body site compared to the total number of clusters containing at least one ORF from this site showed a trend of at least 70%. Only ORFs obtained from lung samples clustered differently with only 50% of lung-containing clusters made exclusively of lung ORFs. The rest of lung-containing clusters were mainly made together with fecal (17%), oral (14%) or both oral and fecal ORFs (11%).

#### Functional Annotation

**FIGURE 3:**
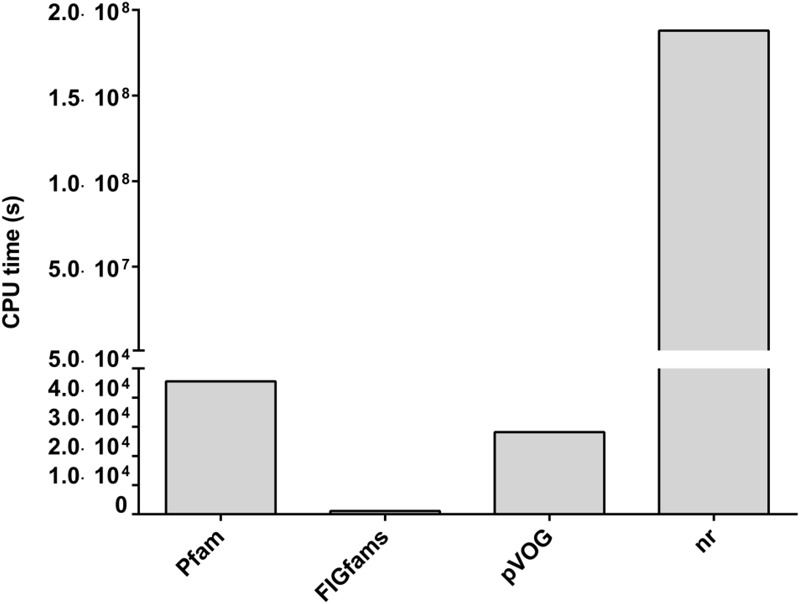
CPU time required for each of the four different methods used for functional annotation of the HVPC database. CPU, central processing unit; pVOG, prokaryotic virus orthologous groups; nr, NCBI’s non-redundant protein database. All jobs were done over a High Performance Computer Cluster (HPCC) in Helmholtz Center Munich using Grid Engine (2011.11) for job distribution. The HPCC is made up of 21 computing nodes: 19 nodes, each with 24 cores (Intel^®^ Xeon^®^ CPU X5690 @ 3.47GHz) and another two nodes, each with 32 cores (Intel^®^ Xeon^®^ CPU E5-2667 v2 @ 3.30GHz).

We used four different databases, nr, Pfam, FIGfams (SEED server) and pVOG, for the functional annotation of HVPC clusters. Notably, a different set of clusters with varying degrees of overlap could be annotated (**Figure 3**). Expectedly, the use of nr allowed the annotation of the highest number of clusters (460,270 clusters equivalent to 49.7%), followed by Pfam and FIGfams, with similar number of clusters annotated (196,316 (21.2%) and 194,936 (21.0%) clusters, respectively), and finally pVOG with only 49,767 (5.4%) clusters annotated. The overall number of clusters annotated with at least one database is 469,101 (50.6%) clusters.

Noticeably, there is a great difference in the central processing unit (CPU) time required to accomplish each of the annotation jobs (**Figure 3**). CPU time ranged from as low as 1,157 s in the case of FIGfams to 1.88 × 10^9^ s for nr; that is more than six orders of magnitude higher. Hmmsearch based annotation in the case of Pfam and pVOG required 45,605 and 28,203 s of CPU time, respectively, which is in the range of one order of magnitude higher than FIGfams and five orders of magnitude lower than nr.

For the top ten clusters, in terms of cluster size, eight could be annotated by at least one annotation method while only two could not be annotated using the selected parameters (**Table 1**). Interestingly, the largest cluster was annotated as beta-lactamase, while the rest were structural proteins (e.g., phage tail), DNA-related proteins (e.g., primase or helicase), beta-galactosidase or proteins of poorly defined function of certain viruses (e.g., Torque Teno Virus or crAssphage).

**Table 1:** Annotation of the top ten HVPC clusters.

							SEED	SEED
Cluster	Size^∗^	nr	Pfam	FIGfams	pVOG	GO	subsystem	category
1	72340	MULTISPECIES: class A broad-spectrum beta-lactamase TEM-116 [Bacteria]	Beta-lactamase	Beta-lactamase (EC 3.5.2.6)	–	–	Virulence, Resistance to antibiotics and toxic compounds	–
2	27442	ORF1 [Torque teno virus]	TT viral orf 1	–	–	–	–	–
3	21304	ORF1 [Torque teno virus]	TT viral orf 1	–	–	–	–	–
4	12799	orf00052, partial [uncultured crAssphage]	–	–	–	–	–	–
5	12425	putative helicase [Lactococcus phage 1706]	–	–	Bbp29; D5-like protein; DNA primase; DNA primase/helicase; DNA primase/polymerase; P4 family phage/plasmid primase; gp108; gp109; gp11; gp34; gp49; gp60; gp68; gp69; gp70; gp71; gp72; gp74; gp81; gp86; gp88; gp89; gp90; gp92; gp94; gp9a; helicase; hypothetical protein; orf40; phage associated primase; phage associated primase/P4 family phage/plasmid primase; phage-associated primase/P4 family phage/plasmid primase; primase; primase/polymerase; putative DNA primase; putative DNA-polymerase or DNA-primase; putative P4 family primase; putative predicted product; putative primase; putative primase/helicase protein	–	–	–
6	11731	–	–	–	–	–	–	–
7	9611	ORF1 [Torque teno virus]	TT viral orf 1	–	–	–	–	–
8	8814	–	–	–	–	–	–	–
9	7169	beta-galactosidase alpha-peptide [unidentified cloning vector]	–	–	–	–	–	–
10	6489	phage tail protein [[Clostridium] symbiosum]	–	–	DNA-binding domain protein; HTH DNA binding domain protein; gp106; hypothetical protein; structural protein	–	–	–

*nr, NCBI’s non-redundant protein database; pVOG, prokaryotic virus orthologous groups; GO, gene ontology; TT, Torque Teno Virus. ^∗^Cluster size refers to the number of ORFs in each cluster.*

### BAL Samples

#### Functional Annotation

**FIGURE 4:**
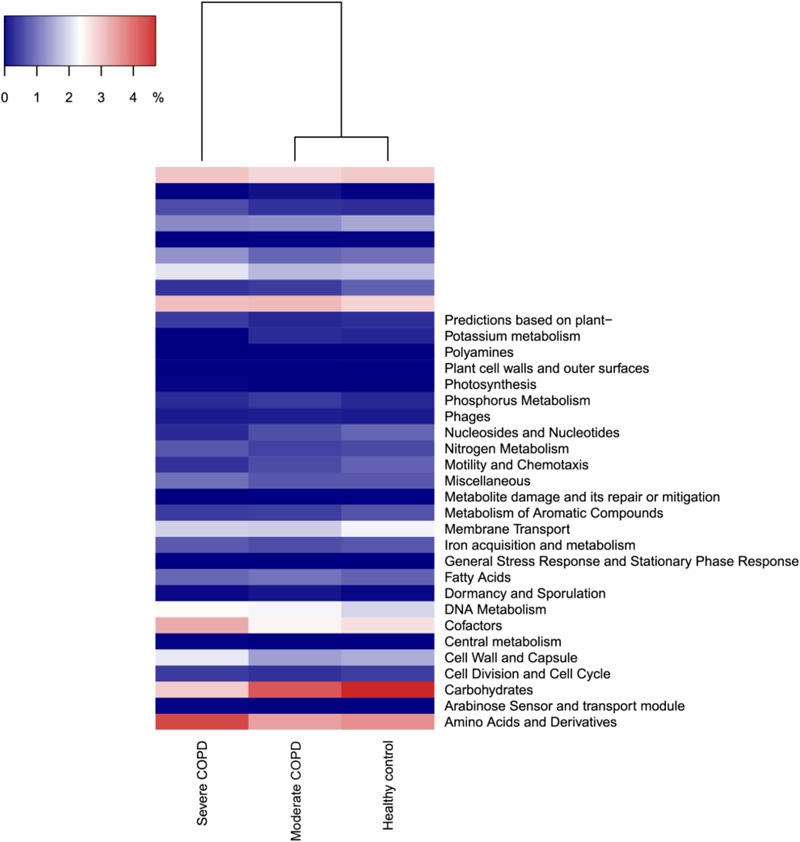
Dendrogram showing the clustering of the three BAL samples based on the abundance of identified SEED categories. COPD, chronic obstructive pulmonary disease. The figure was generated in R v3.4.0 (R Core Team, 2017) using heatmap3 v1.1.1 (Zhao et al., 2015).

**FIGURE 5:**
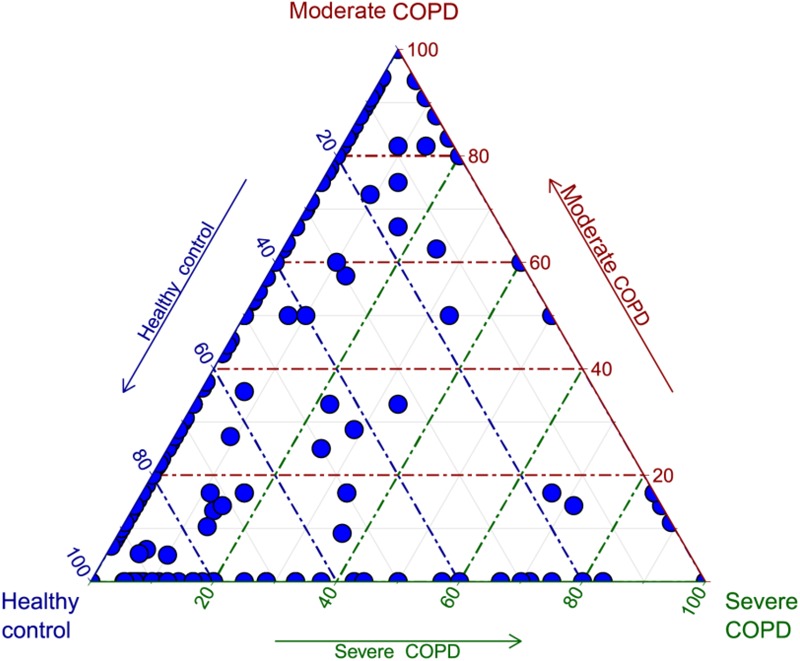
Triagonal plot showing, for each cross-assembled contig, the percentage of contributing reads from each of the three BAL samples. Each contig is represented by a small blue circle. The plot was generated in R v3.4.0 (R Core Team, 2017) using ggtern v2.2.1 (Hamilton, 2017).

As an application for the use of HVPC, we aligned ORFs called from three BAL samples collected from one healthy individual and two COPD patients (moderate and severe) to the HVPC database for functional annotation. On average, 64% of BAL sequences could be aligned to HVPC and almost all of them could be functionally annotated (**Table 2**). In contrast, around 9% of sequences could be aligned to protein-coding sequences from RefSeq and non-RefSeq full genome DNA viruses in NCBI. Strikingly, only 10% of these sequences could be functionally annotated because most proteins were hypothetical or of unknown function. Remarkably, annotation and classification of ORFs into SEED categories allowed the clustering of healthy and moderate COPD BAL samples together (**Figure 4**). The most abundant SEED categories observed in the three samples were carbohydrate metabolism, amino acids and derivatives, protein metabolism and virulence. Regardless of the annotation, the healthy control and the moderate COPD samples showed higher similarity in terms of the number of shared HVPC clusters (**Supplementary Figure S1**). Likewise, comparative analysis of the three viromes in a reference-independent way, using crAss, showed that the healthy control and the moderate COPD samples had more similarity to one another. Generally, the cross-assembly was made out of 1,111 contigs (**Figure 5**). Only 32 contigs were shared between the three BAL samples (dots lying in the middle of the triangle plot). Contigs shared only between pairs of samples (represented by dots lying on the edges between each pair in the triangular plot) were as follows: 198 contigs shared between the healthy control and the moderate COPD, 68 shared between the healthy control and the severe COPD and only 18 shared between the moderate COPD and the severe COPD. The remaining contigs (represented by dots at the triangle vertices) were unique for each sample: 559 contigs for the healthy control, 229 for the moderate COPD and 15 for the severe COPD.

**Table 2:** Comparison of functional annotation using HVPC versus RefSeq and non-RefSeq DNA viruses.

Dataset	#Sequences	#Sequences aligned to HVPC	% aligned	Functionally annotated	% annotated	#Sequences aligned to RefSeq and non-RefSeq DNA viruses	% aligned	Functionally annotated	% annotated
Healthy control	27669	18425	66.6%	18212	65.8%	2941	10.6%	318	1.1%
Moderate COPD	8956	5546	61.9%	5541	61.9%	719	8.0%	61	0.7%
Severe COPD	2522	1619	64.2%	1604	63.6%	226	9.0%	21	0.8%

#### Taxonomic Classification

**FIGURE 6:**
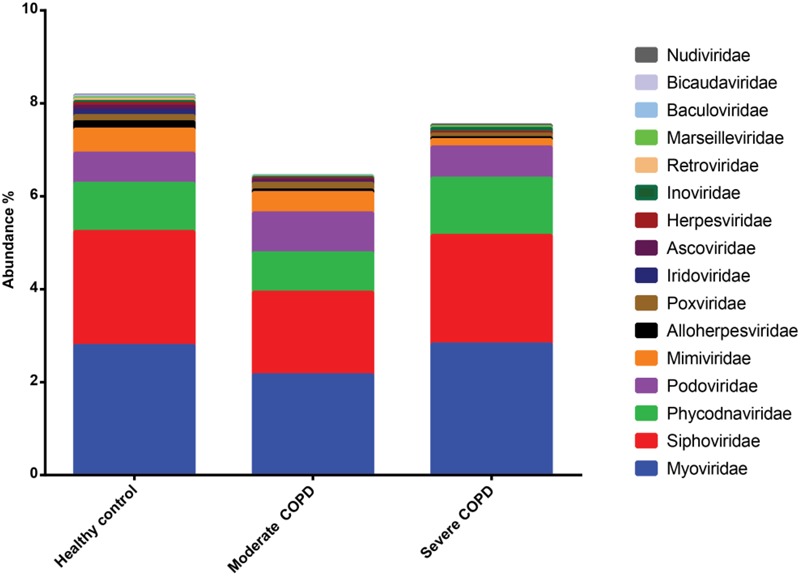
Abundance of viral families identified in BAL samples. Families with abundances less than 0.01% in all samples were removed.

Approximately 90% of sequences in the three BAL samples could not be assigned to any known viral lineage. For identified sequences, the most abundant viral family was Myoviridae followed by Siphoviridae, Phycodnaviridae and Podoviridae (**Figure 6**). The remaining viral families had abundances of less than or equal to 0.05%. Both prokaryotic and eukaryotic DNA viruses could be identified. Concerning prokaryotic viruses, the abundance of phages was variable between samples, yet Bacillus virus G was the most abundant among all samples (**Supplementary Figure S2**). Interestingly, few archaeal viruses e.g., Halovirus HGTV-1 and Natrialba phage PhiCh1) could also be detected. Of note, the abundance of certain phages whose hosts are common in COPD was found to be generally higher in COPD samples compared to the healthy control (**Table 3**). The abundance of eukaryotic viruses was also variable (**Supplementary Figure S3**) with Megavirus chilensis being the most abundant in the healthy control and the moderate COPD samples, while for the severe COPD sample, the most abundant eukaryotic virus was Paramecium bursaria Chlorella virus A1. Again, the abundance profiles of common eukaryotic and prokaryotic viruses allowed the clustering of healthy control and moderate COPD samples together (**Supplementary Figures S2, S3**). Noteworthy, some viral species (67 for moderate COPD and 24 for severe COPD) could be found only in COPD samples and not in the healthy control (**Supplementary Table S3**). This flexible virome showed higher richness for the moderate COPD compared to the COPD sample.

**Table 3:** Abundance of selected phages whose hosts are commonly found in COPD.

Virus^∗^	Healthy control	Moderate COPD	Severe COPD
Bordetella phages	nd	0.07%	0.04%
Human alphaherpesvirus 1	nd	nd	0.04%
Moraxella phages	0.15%	0.12%	0.32%
Pseudomonas phages	0.29%	0.40%	0.36%
Stenotrophomonas phages	0.02%	0.03%	0.08%
Streptococcus phages	0.15%	0.39%	0.40%

*^∗^All virus infecting the same host. nd, not detected.*

#### Diversity

Diversity was assessed on both the functional and the taxonomic levels. Functional diversity was determined in an annotation-independent way, based on the number of different HVPC clusters to which BAL sequences could be assigned in each sample, irrespective whether a cluster could be functionally annotated or not. None of the samples reached rarefaction (**Supplementary Figure S4**), but generally it can be observed that functional diversity is highest in the healthy control sample and lowest in the severe COPD (**Table 4**). The same can be noticed for taxonomic diversity, where samples could not reach rarefaction as well (**Supplementary Figure S5**), nevertheless diversity was highest in the case of the healthy control sample, followed by the moderate COPD and the severe COPD samples, respectively (**Table 4**).

**Table 4:** Functional and taxonomic diversity of the three BAL samples.

	Taxonomic diversity				Functional diversity			
		Inverse		Species		Inverse		Species
Sample	Shannon	simpson	Fisher’s α	richness	Shannon	simpson	Fisher’s α	richness
Healthy control	5.48	97.23	230.32	604.00	8.94	5206.20	9180.23	10107.00
Moderate COPD	5.00	84.29	131.02	245.00	7.96	2255.32	3879.90	3444.00
Severe COPD	4.42	52.12	103.81	120.00	6.94	871.69	1893.53	1170.00

#### Virome-Borne Virulence Factors

**FIGURE 7:**
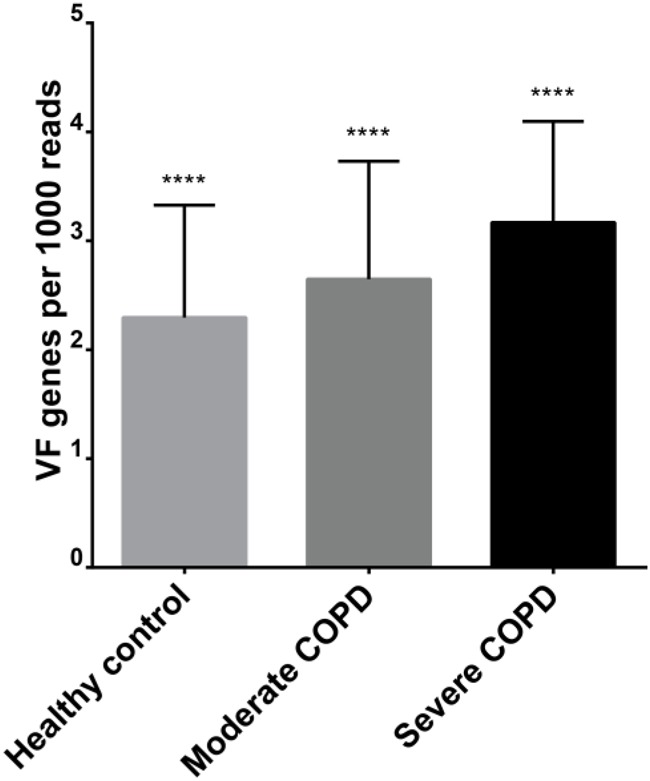
Relative abundance of virulence factors in each of the three BAL viromes. ^∗∗∗∗^ denote statistical significant difference with a *p*-value of less than 0.0001.

We evaluated the possibility that the studied viromes could harbor potential bacterial virulence factors. For this purpose, BAL sequences were aligned to polypeptides in the core VFDB. Expectedly, the severe COPD virome sample had the highest relative abundance of bacterial virulence factors followed by the moderate and the severe COPD samples, respectively (**Figure 7**). Comparison of the means of relative abundances after 10,000 rounds of random subsampling showed statistical significance through ANOVA (*p*-value < 0.0001). Besides, all sets of pairs showed statistically significant difference from each other (*p*-value < 0.0001). In all samples, the most represented virulence factors had resemblance to those of *Pseudomonas aeruginosa* e.g., components of xcp, type IV, and type VI secretion systems, pyoverdin and components of alginate biosynthesis and O-acetylation (for biofilm formation) (**Supplementary Table S4**).

## Discussion

In this study, we built a protein cluster database, the HVPC, from 244 human virome datasets with the aim of providing the scientific community with a new resource that could help attain a better functional annotation of human viromes. Actually, the use of protein clusters with a similar purpose was first done on the microbiome level in the Global Ocean Sampling (GOS) study (Rusch et al., 2007). Later, the same concept was adopted for several environmental virome datasets e.g., the Pacific Ocean Virome (POV) (Hurwitz and Sullivan, 2013), the Tara Oceans Virome (TOV) (Brum et al., 2015) and the Earth Virome (Paez-Espino et al., 2016). Yet, to our knowledge, this is the first time this concept is employed for human viromes. The HVPC was built from the viromes of six body sites. Interestingly, the core human virome, that is the virome shared between all body sites, was minimal. In only nine clusters out of 390,917 (0.002%), sequences from the six different sites were represented (**Figure 2A**). Functional annotation of these protein clusters showed that they generally contribute to core functions e.g., DNA metabolism, transcription, translation and protein folding (**Supplementary Table S5**). In contrast, most of the HVPC clusters were made out of sequences from a single site and the number of clusters with ORFs from more than one site was marginal. This habitat-dependent composition was similarly reported for the human microbiome (The Human Microbiome Project Consortium, 2012; Chu et al., 2017), where both the structure and function of microbiota were site-specific. In contrast, ORFs originating from lung samples had a different clustering pattern in which approximately half of the clusters containing lung ORFs did include ORFs from other body sites, especially gut and mouth. This observation could be explained by hypothesizing that the lung virome follows a similar pattern to that of the lung microbiome. Indeed, the oral microbiome is believed to be the main source for the bacterial community in the lungs (Dickson and Huffnagle, 2015). Similarly, it is expected to find some of the microbial communities shared between the gut and the lungs since (i) ingested microbes have the ability to access both sites and (ii) microbes from the gut can enter the respiratory tract through aspiration (Budden et al., 2017).

For annotation of the protein clusters, we chose to implement four different methods to obtain as much information about these clusters as possible and we leave it to the users of the database to select the method they find most suitable or integrate information from all methods. Each method gave a different set of annotations. Although they do intersect, each method still annotated some clusters that could not be annotated by the others. The most comprehensive annotation was obtained by nr probably because of its huge size (120,095,048 sequences as of April 18, 2017). However, this huge size combined with the use of BLAST for alignment led to the slowest performance. Following nr, Pfam and FIGfams gave more or less similar level of annotation, but FIGfams was much faster presumably because (i) although FIGfams annotation was submitted from the Helmholtz Center Munich HPCC, the process actually ran on the SEED server that is obviously more powerful and (ii) FIGfams annotation relies on a BLAST voting procedure that was shown to be faster and independent on the number of queried families when compared to HMM-based search (Meyer et al., 2009). The lowest level of annotation was obtained using the pVOG, possibly because these orthologous groups were generated from prokaryotic viruses only (Grazziotin et al., 2017), while the HVPC is expected to contain proteins from eukaryotic viruses as well.

Of note, the largest HVPC cluster was annotated as beta-lactamase. This finding highlights the role of viromes in the spread of antibiotic resistance through horizontal gene transfer. Indeed, beta-lactamases as well as other resistance genes were previously detected in the human gut virome (Minot et al., 2011) and in CF patients sputum viromes (Fancello et al., 2011). Nevertheless, it has recently been claimed that the number of antibiotic resistance genes detected in viromes is overestimated and that experimental evidence fails to confirm that the detected genes can confer resistance (Enault et al., 2017). Another enzyme, beta-galactosidase, was also detected among the top ten clusters. This enzyme is usually used by bacteria to ferment lactose, but one can easily find some examples for beta-galactosidases in bacteriophages (e.g., NCBI accessions YP_009006046 and YP_007003234.1 in *Klebsiella* phage F19 and *Lactobacillus* phage LF1). This probably means that during viral infection, virus-derived copies of this enzyme are expressed in order to enhance bacterial fitness. This phenomenon was previously shown for photosynthesis genes in marine phages (Lindell et al., 2005). In order to demonstrate the usefulness of the HVPC, we used it for the functional annotation of three BAL samples, one from each of a healthy control, a moderate and a severe COPD patients. We could show that the use of the HVPC allowed a seventy-fold increase in the annotation level compared to the case when only limited to information from full genome DNA viruses. Even in the absence of annotation information from the clusters, alignment to the HVPC can still be informative, as they can give an indication about the diversity of the aligned samples. For the BAL samples, although they did not reach rarefaction, all diversity indices inferred from alignment to the HVPC point to a diversity that is highest in the healthy control sample and is reduced in the COPD samples according to case severity. This pattern coincides with that obtained from the taxonomic diversity analysis. Indeed, this disease-linked dysbiosis is common for the normal microbiota in many diseases (Belizário and Napolitano, 2015). The diversity of the lung virome was similarly reported to be greatly reduced in the highly diseased lobes of an explanted lung from a late-stage CF patient (Willner et al., 2012). Besides, by utilizing the HVPC, we could show that the healthy control and moderate COPD viromes were more similar to each other than to the severe COPD virome. This similarity was shown in both annotation-dependent and independent ways. Specifically, annotations for each virome grouped into SEED categories allowed the clustering of the healthy control and moderate COPD viromes together. In addition, the number of HVPC hit clusters shared between the healthy control and moderate COPD viromes again showed higher similarity. This pattern was confirmed by cross-assembly in a reference-independent way and by taxonomy results, which allowed the clustering of the healthy and moderate COPD pair together, based on shared prokaryotic and eukaryotic viruses.

On the taxonomic level, the majority of virome sequences usually fail to be assigned taxonomy (Willner et al., 2009; Hurwitz and Sullivan, 2013), and BAL viromes in this study were not an exception. Nevertheless, information content from known sequences might still allow better understanding of COPD disease progression. Actually, it seems that there is a core lung virome that shows different dynamics with progressive disease severity. Indeed, it was previously shown that the respiratory tract possesses a core virome for both prokaryotic and eukaryotic viruses (Willner et al., 2009). Some of the core eukaryotic viruses reported by Willner and colleagues in this CF virome study (Willner et al., 2009) have been detected in our studied BAL viromes e.g., *Acanthamoeba polyphaga mimivirus, Ectocarpus siliculosus* virus 1 and Paramecium bursaria Chlorella virus. These eukaryotic viruses generally belong to Phycodnaviridae and Mimiviridae, which usually infect marine or freshwater algae (Wilson et al., 2009) and amoeba (Aherfi et al., 2016), respectively. This observation could support the proposed environmental influence on the lung virome (Willner et al., 2009). Indeed, a mimivirus was previously isolated from a pneumonia patient (Saadi et al., 2013). This virus showed sequence similarity to Megavirus chilensis, which is the most abundant eukaryotic virus in the healthy control and the moderate COPD samples. The isolation of this virus from a pneumonia patient does not necessarily mean pathogenicity and any claim of pathogenicity still warrants further study. In contrast, the prokaryotic viruses in the CF study (Willner et al., 2009) showed no similarity to those identified in our BAL samples. This disagreement could be attributed in part to the differences in databases, especially for the prokaryotic viruses, because authors used a custom database of only 510 phage genomes. Another explanation could be environmental differences as evidently, the inhaled air influences the composition of lung viromes and microbiomes (Willner et al., 2009; Li et al., 2017). On the other hand, COPD samples contained some viruses that were not shared with the healthy control. This flexible virome had higher species richness in the moderate COPD, an observation that confirms the progressive dysbiosis that accompanies disease development. In fact, some viruses that are directly associated with COPD and its exacerbation e.g., human alphaherpesvirus 1 (also known as herpes simplex virus 1) (McManus et al., 2009) or whose hosts are commonly isolated from COPD patients e.g., *Bordetella, Moraxella, Pseudomonas Stenotrophomonas* and *Streptococcus* (Murphy, 2008; Brooke, 2012; King et al., 2013; Hashemi et al., 2015) have been found to be of higher abundance or exclusively present in COPD samples (**Table 3**).

Further analysis showed that BAL viromes could harbor potential bacterial virulence factors. The abundance of these factors increases with increasing COPD disease severity (**Figure 7**). Truly, phages, which constitute the major fraction of most viromes, do play a crucial role in the horizontal gene transfer of bacterial genes. Actually, the expression and dissemination of many bacterial toxins and virulence factors rely on phages [reviewed in (Penadés et al., 2015)].

## Conclusion

In conclusion, we have built a HVPC that could be a useful resource for better annotation and characterization of human viromes. To our knowledge, this is the first database exclusively focusing on human viromes. The database proved useful in the functional annotation of our BAL virome samples, which are otherwise poorly characterized when limited to annotation using sequences from full-length viral genomes. In addition, our BAL samples gave a first insight into viral communities of COPD patients and confirm a state of dysbiosis for viruses that increases with disease progression. Moreover, they shed light on the potential role of phages in the horizontal gene transfer of bacterial virulence factors, a phenomenon that highlights a possible contribution of phages to etiopathology.

## Data Availability

The HVPC and related functional annotations are available through the following link: https://osf.io/gs4zf/ (doi: 10.17605/OSF.IO/GS4ZF).

## Author Contributions

LD, AE, JF, and CG conceived the idea for the manuscript. AE, JF, and DS performed the experiment and analyzed the data. AE, JF, and LD wrote the manuscript.

## Conflict of Interest Statement

The authors declare that the research was conducted in the absence of any commercial or financial relationships that could be construed as a potential conflict of interest.

## Footnotes

1 https://www.ncbi.nlm.nih.gov/sra

2 http://metagenomics.anl.gov/

3 http://www.mgc.ac.cn/VFs/download.htm

## Supplementary Material

FIGURE S1 Venn diagram showing the number of shared HVPC clusters between the three BAL samples.

FIGURE S2 Heat map showing the relative abundance of 36 prokaryotic viruses shared between the three BAL samples.

FIGURE S3 Heat map showing the relative abundance of 15 eukaryotic viruses shared between the three BAL samples.

FIGURE S4 Rarefaction curves showing the number of Human Virome Protein Cluster (HVPC) hits versus sample size based on random subsampling and a step size of 50 sequences.

FIGURE S5 Rarefaction curves showing the number of species versus sample size based on random subsampling and a step size of 50 sequences.

TABLE S1 Metadata of downloaded virome datasets.

TABLE S2 Basic statistics of downloaded metageomes before and after quality control.

TABLE S3 The flexible virome of COPD samples.

TABLE S4 Blast results for BAL sequences vs the core Viruelence Factors Database.

TABLE S5 Functional annotation of the representative ORFs of the core human virome clusters.
