# Supplementary material for: The Human Virome Protein Cluster Database (HVPC): A Human Viral Metagenomic Database for Diversity and Function Annotation
Source: Front Microbiol. 2018 May 29;9:1110. doi: 10.3389/fmicb.2018.01110 (PMC5987705; doi:10.3389/fmicb.2018.01110)
Supplement: FIGURE S1 — Venn diagram showing the number of shared HVPC clusters between the three BAL samples. [file Image_1.PDF]

Supplementary Figures

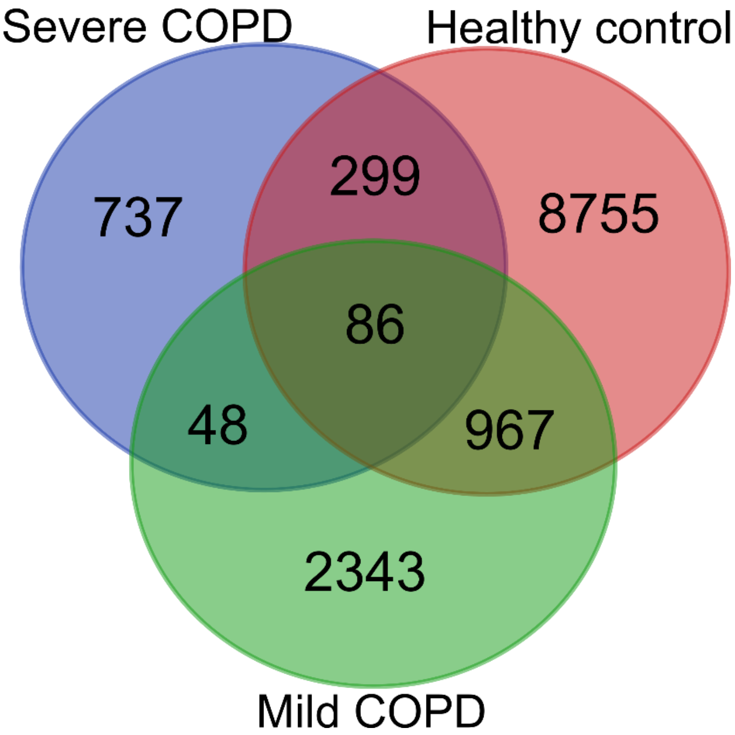


Figure S1. Venn diagram showing the number of shared HVPC clusters between the three BAL samples.

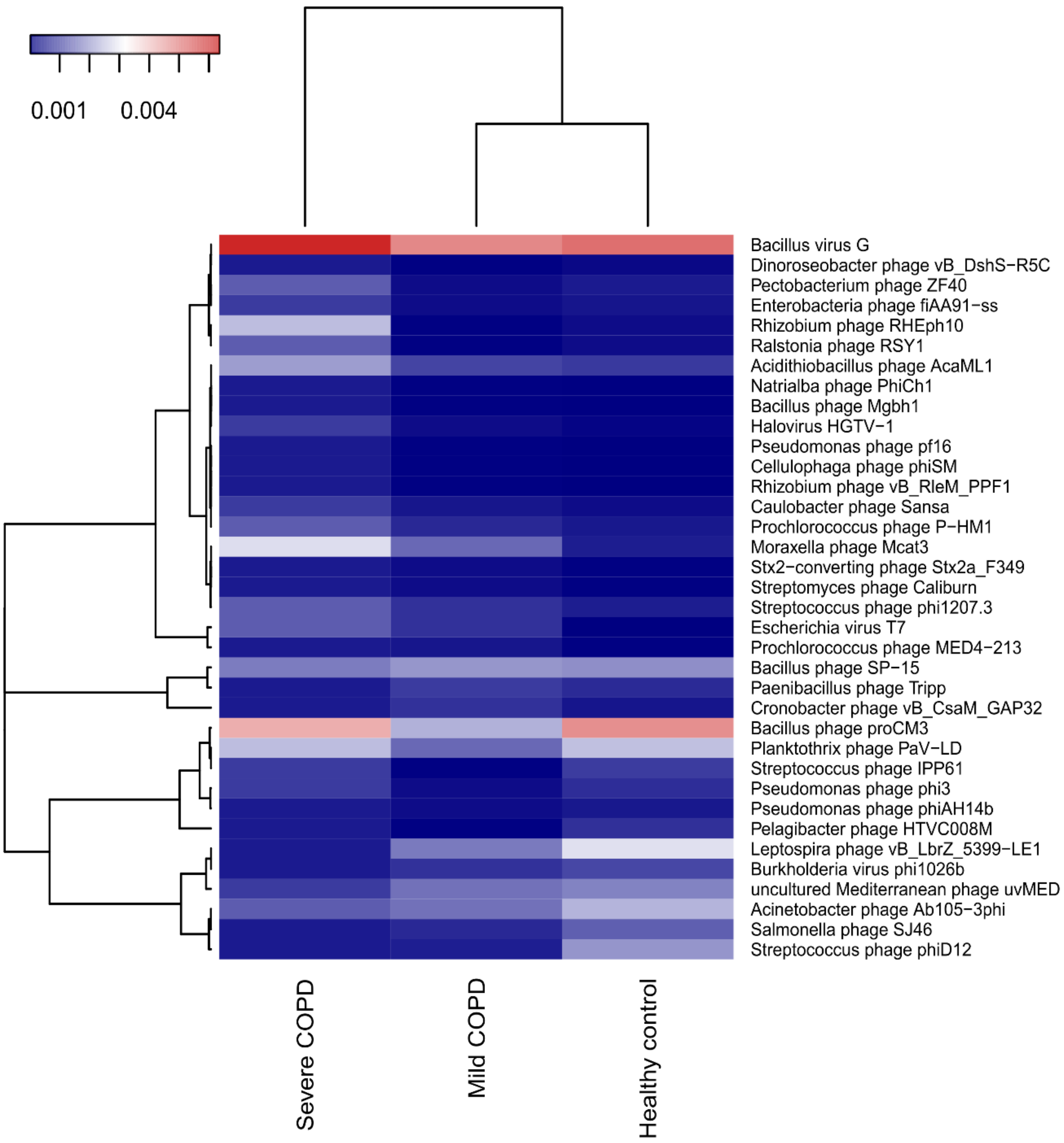


Figure S2. Heat map showing the relative abundance of 36 prokaryotic viruses shared between the three BAL samples.

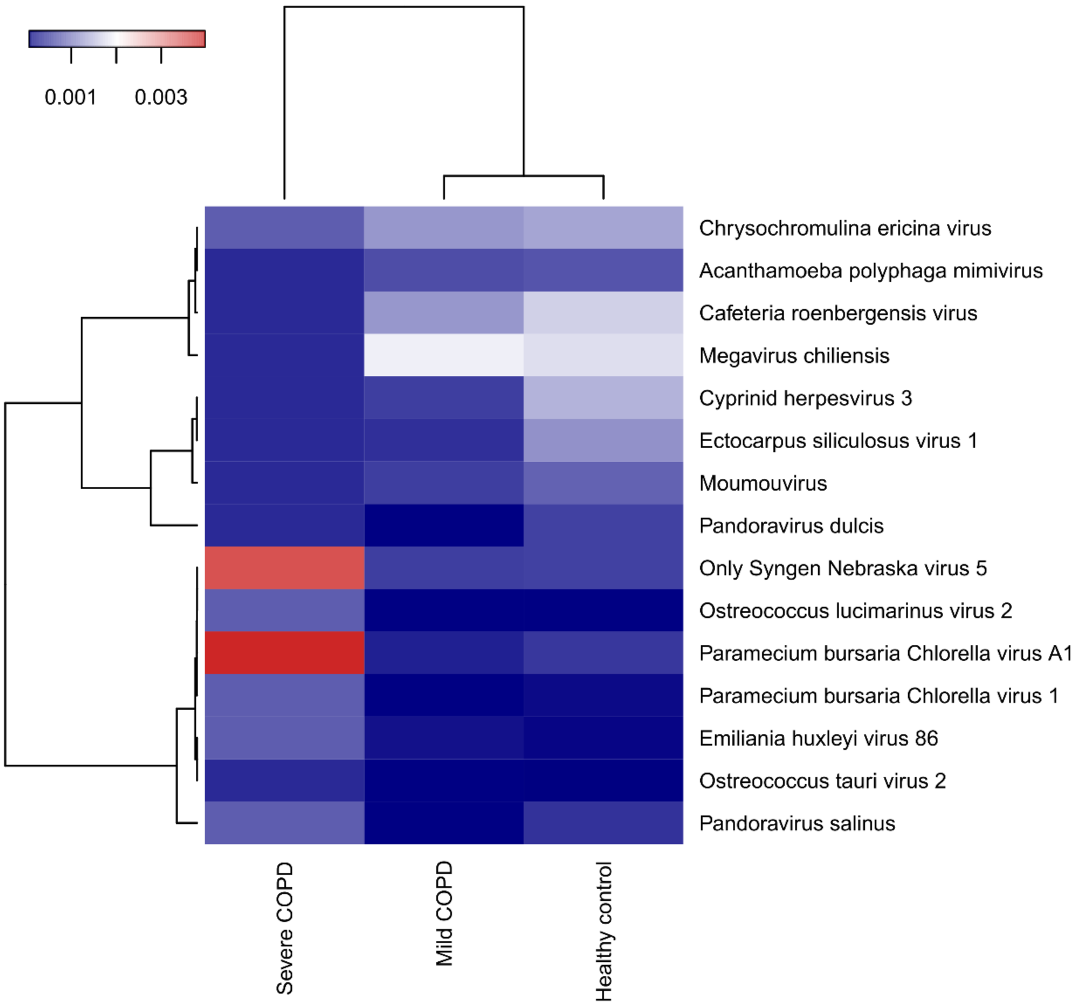


Figure S3. Heat map showing the relative abundance of 15 eukaryotic viruses shared between the three BAL samples.

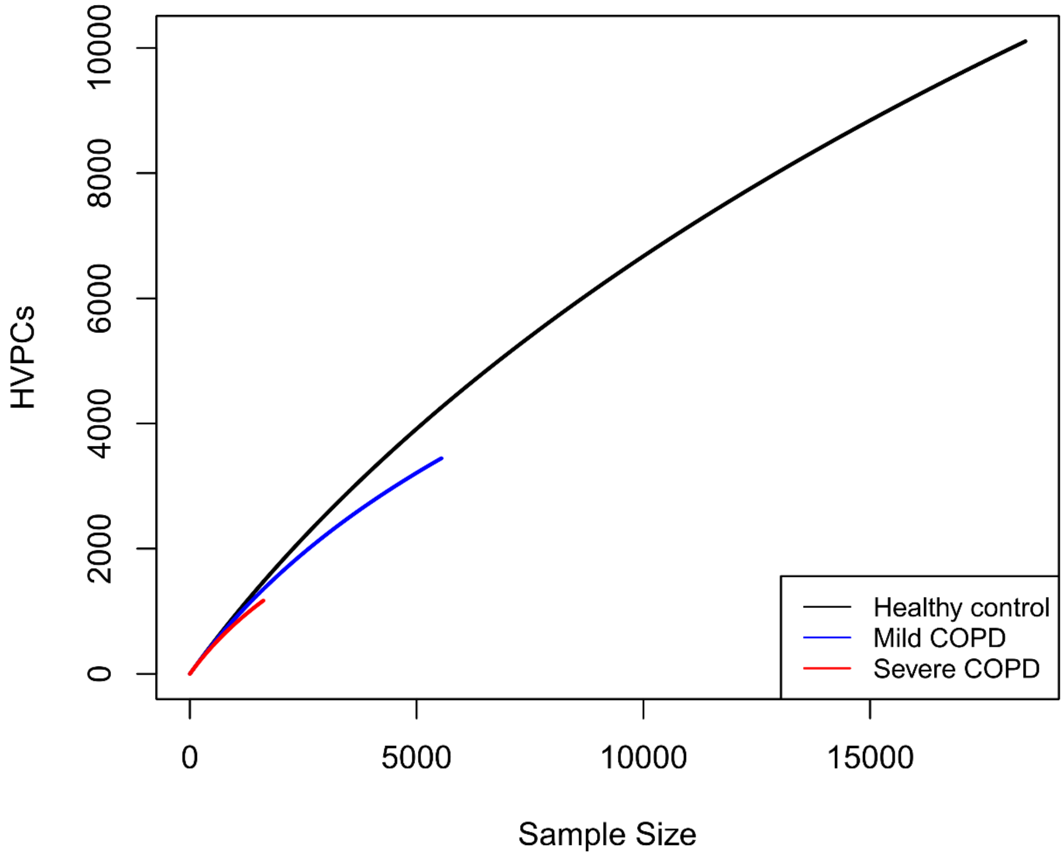


Figure S4. Rarefaction curves showing the number of Human Virome Protein Cluster (HVPC) hits versus sample size based on random subsampling and a step size of 50 sequences.

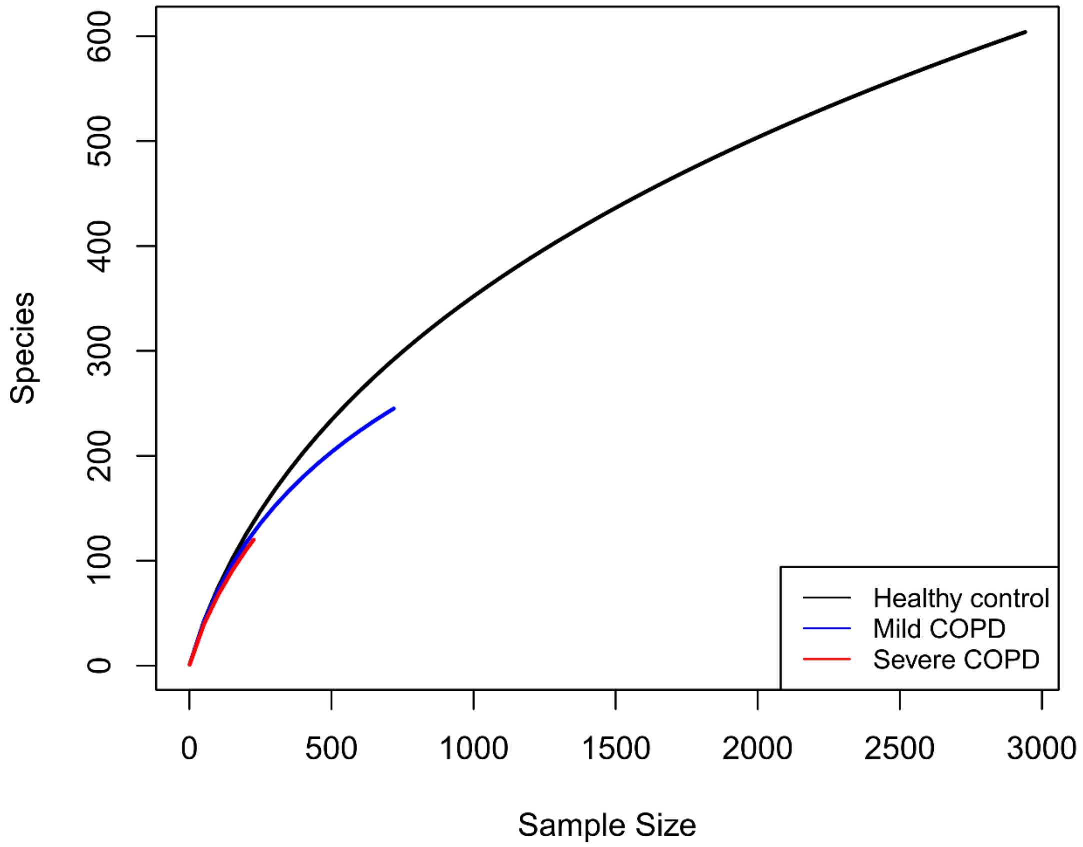


Figure S5. Rarefaction curves showing the number of species versus sample size based on random subsampling and a step size of 50 sequences.
